# The Clinical Management of Electrical Stimulation Therapies in the Rehabilitation of Individuals with Spinal Cord Injuries

**DOI:** 10.3390/jcm13102995

**Published:** 2024-05-20

**Authors:** David R. Dolbow, Ines Bersch, Ashraf S. Gorgey, Glen M. Davis

**Affiliations:** 1 Physical Therapy Program, College of Osteopathic Medicine, William Carey University, 710 William Carey Parkway, Hattiesburg, MS 39401, USA; 2 International FES Centre®, Swiss Paraplegia Centre, CH-6207 Nottwil, Switzerland; 3 Spinal Cord Injury and Disorders Center, Hunter Holmes McGuire VA Medical Center, Richmond, VA 23249, USA; ashraf.gorgey@va.gov; 4 Discipline of Exercise and Sport Sciences, Sydney School of Health Sciences, Faculty of Medicine and Health, The University of Sydney, Camperdown, NSW 2006, Australia

**Keywords:** neuromuscular electrical stimulation, functional electrical stimulation, spinal cord injury, tenodesis effect

## Abstract

**Background:** People with spinal cord injuries (SCIs) often have trouble remaining active because of paralysis. In the past, exercise recommendations focused on the non-paralyzed muscles in the arms, which provides limited benefits. However, recent studies show that electrical stimulation can help engage the paralyzed extremities, expanding the available muscle mass for exercise. **Methods:** The authors provide an evidence-based approach using expertise from diverse fields, supplemented by evidence from key studies toward the management of electrical stimulation therapies in individuals with SCIs. Literature searches were performed separately using the PubMed, Medline, and Google Scholar search engines. The keywords used for the searches included functional electrical stimulation cycling, hybrid cycling, neuromuscular electrical stimulation exercise, spinal cord injury, cardiovascular health, metabolic health, muscle strength, muscle mass, bone mass, upper limb treatment, diagnostic and prognostic use of functional electrical stimulation, tetraplegic hands, and hand deformities after SCI. The authors recently presented this information in a workshop at a major rehabilitation conference. Additional information beyond what was presented at the workshop was added for the writing of this paper. **Results:** Functional electrical stimulation (FES) cycling can improve aerobic fitness and reduce the risk of cardiovascular and metabolic diseases. The evidence indicates that while both FES leg cycling and neuromuscular electrical stimulation (NMES) resistance training can increase muscle strength and mass, NMES resistance training has been shown to be more effective for producing muscle hypertrophy in individual muscle groups. The response to the electrical stimulation of muscles can also help in the diagnosis and prognosis of hand dysfunction after tetraplegia. **Conclusions:** Electrical stimulation activities are safe and effective methods for exercise and testing for motor neuron lesions in individuals with SCIs and other paralytic or paretic conditions. They should be considered part of a comprehensive rehabilitation program in diagnosing, prognosing, and treating individuals with SCIs to improve function, physical activity, and overall health.

## 1. Introduction

Individuals with spinal cord injuries (SCIs) and other paralytic or paretic conditions often face challenges in maintaining their health and mobility due to reduced physical activity [1,2,3,4,5]. A host of comorbidities develop from a combination of the neuropathology of the injury and the decreased physical activity levels associated with the injury [6]. Common comorbidities include cardiometabolic conditions such as neurogenic obesity [7,8,9], metabolic syndrome [10,11], cardiovascular complications [12,13,14] including orthostatic hypotension [15,16] and autonomic dysreflexia [17,18]. Early recommendations for exercise after SCI suggested voluntary exercise with the non-paralyzed muscles of the arms, which limited the activity workload due to the reduced amount of available active skeletal muscle [19,20,21]. However, recent scientific research has demonstrated the benefits of electrical stimulation-evoked exercise, leading to the recommendation of neuromuscular electrical stimulation (NMES) resistance training and functional electrical stimulation (FES) cycling for individuals with SCIs [22,23].

NMES involves using electrical impulses to stimulate the paralyzed muscles, inducing muscle contractions, and increasing the range of physical activities that can be performed. This includes resistance training, which can enhance muscle strength, endurance, and power [24,25,26,27,28,29]. FES exercises, such as cycling, also use electrical impulses to stimulate the affected muscles, enabling the individual to engage in physical activities that would otherwise be impossible [30,31,32,33].

The review sought to summarize important advancements in NMES and FES interventions for individuals with SCIs. Through an analysis of studies, this review showcases evidence supporting the use of these interventions for enhancing lean mass volume; improving cardiovascular and metabolic outcomes; potentially reducing bone loss; and diagnosing, prognosing, and treating hand dysfunction in this population.

## 2. Methods

The authors used the evidence-based process of combining their expertise from diverse fields supplemented by separate scientific literature searches for key evidence related to the management of electrical stimulation therapies in the rehabilitation of individuals with SCIs. The search engines used for the literature searches included PubMed, Medline, and Google Scholar. The keywords used for the separate searches included functional electrical stimulation cycling, neuromuscular electrical stimulation resistance training, spinal cord injury, cardiovascular health, metabolic health, muscle strength, muscle mass, bone mass, upper limb treatment, and diagnostic and prognostic use of functional electrical stimulation for the hands of those with tetraplegia. The inclusion criteria included articles involving individuals with SCIs; the use of electrical stimulation for treatment, diagnosis, or prognosis; and outcomes related to cardiovascular health, metabolic health, muscle strength, muscle mass, bone mass, and upper limb function. The exclusion criteria included articles 20 years or older and those that did not match the inclusion criteria. The authors recently presented this information in a workshop at a major rehabilitation conference. Additional information beyond what was presented at the workshop was added for the writing of this paper. Individuals that suffer a traumatic SCI undergo an initial rapid decline in muscle mass and strength and bone mass. For this reason, we focused on research that attempted to regain lost muscle and bone a year or more post-injury after muscle atrophy and bone demineralization had slowed. Thus, for the topics of muscle, bone, and cardiometabolic health, we focused on chronically injured individuals (>1 year post injury). For the diagnostic and prognostic evaluations for individuals with tetraplegia, the paper focused on more acute SCIs.

## 3. Results

### 3.1. Cardiovascular and Metabolic Health (Table 1)

**Figure 1:**
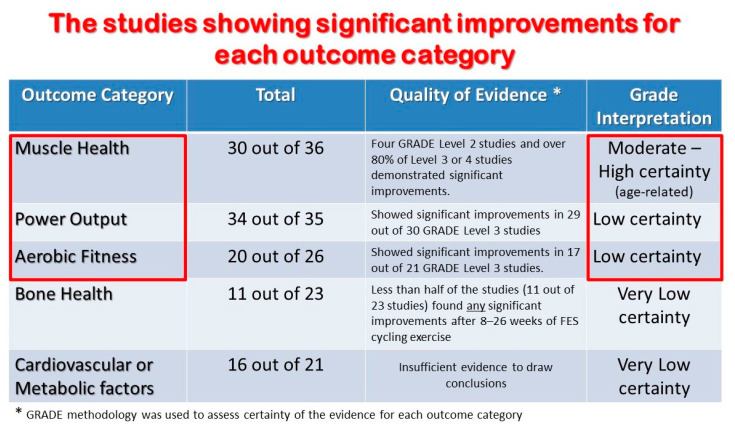
Significant improvements by outcome category (modified from van der Scheer et al. [34], reprinted from Street and Davis Forward, [35].

A systematic review of research by van der Scheer and colleagues [34] found that 30 out of 36 peer-reviewed studies provided moderate to high evidence supporting the effectiveness of FES cycling in improving muscle health if performed for 30 min, three times a week for 16 or more weeks. These studies applied electrical stimulation settings to maximize power output at 30–50 revolutions per minute cycling cadence. However, there was weaker evidence of whether FES leg cycling activities could provide sufficient ‘dose potency’ to increase power output and aerobic fitness, and the authors gave those health outcomes a ‘low certainty’ GRADE rating (Figure 1).

One randomized controlled trial found that voluntary arm crank exercise (ACE) significantly outperformed FES leg cycling for improvements in peak oxygen utilization (VO_2_peak) [36]. Specifically, FES leg cycling only resulted in a 2.5% increase in VO_2_peak, compared to an over 20% increase achieved through ACE. Similarly, a separate study found that FES leg cycling was less effective than ACE, hybrid cycling (FES legs cycling plus ACE), and outdoor arm and leg cycling in reaching training levels to improve VO_2_peak [37]. However, upon the re-analysis and speculation of the exercise intensity required to achieve a cardiovascular training effect for low-aerobic-fitness-conditioned individuals (such as individuals with tetraplegia, elderly individuals, or morbidly obese individuals), it was hypothesized that it is possible that FES leg cycling could lead to improvements in cardiovascular fitness in these low-fitness clinical populations [38]. Nonetheless, the authors concluded that hybrid FES cycling usually led to greater cardiovascular fitness improvement due to the higher cardiovascular demand during submaximal exercise.

The aerobic fitness benefits of FES leg cycling were highlighted by Johnston and associates [39] in 30 5-to-13-year-old children with SCIs after performing 40 min of FES leg cycling, passive cycling, or NMES therapy three times per week for six months. They discovered a significant increase in VO_2_peak (16%) with FES leg cycling, while no improvements were observed in VO_2_peak in the passive cycling or NMES therapy groups. However, the NMES therapy group was the only group to show decreased blood cholesterol levels (17%).

Aerobic fitness improvements are typically dependent on workload intensity, so it is reasonable to conclude that hybrid cycling, which combines FES leg cycling with ACE, may provide greater aerobic and cardiovascular health benefits than either FES leg cycling or ACE alone due to the larger muscle mass involved in such exercise. Brurok et al. [40] investigated the effects of hybrid FES cycling thrice weekly for eight weeks. A high-intensity interval training (HIIT) protocol utilized four exercise bouts at 85–90% of maximal workload for ACE and 80% of 140 mA electrical stimulation amplitude for the legs during the four-minute high-intensity exercise bouts. Three minutes of low-intensity exercise (70% of maximal workload for ACE and assisted leg cycling without electrical stimulation) was interspersed with the high-intensity bouts. After eight weeks of hybrid HIIT-FES cycling, the participants realized a 33% increase in stroke volume, a 27% increase in cardiac output, and a 28% increase in VO_2_peak over the exercise-free control period. Similarly, in a separate study, six weeks of hybrid HIIT-FES cycling with virtual-reality feedback produced a 33% increase in power output and a 20% increase in VO_2_peak [41]. However, because blood lipid and glucose levels were unchanged, the authors contemplated whether more than six weeks of hybrid HIIT-FES cycling might be required to show benefits in cardiovascular health blood markers. In this study, eight adults with SCI exercised for 32 min three times per week or 48 min twice weekly, totaling 96 min of hybrid HIIT-FES cycling per week.

A study that combined NMES resistance training with FES leg cycling resulted in higher VO_2_peak levels and reduced visceral adipose tissue. Twelve weeks of NMES resistance training plus twelve weeks of FES leg cycling was compared to twelve weeks of passive leg movement plus twelve weeks of FES leg cycling. The results showed that NMES resistance training plus twelve weeks of FES leg cycling was more effective than passive leg movement therapy followed by FES leg cycling in improving VO_2_peak levels, with respective increases of 29% and 16% [42].

In a separate study, Gorgey et al. [43] demonstrated improvements in cardiovascular blood markers with positive lipid changes after 12 weeks of twice-weekly NMES resistance training. Free fatty acid levels decreased by 24%, triglyceride levels decreased by 38%, and the cholesterol/high-density lipoprotein ratio also decreased.

Regarding potential metabolic benefits, Sanchez and associates [44] performed a meta-analysis on nine studies investigating evidence that NMES effectively improves glycemic control predominantly in a middle-aged and elderly population with type-2 diabetes, obesity, and SCI. The meta-analysis showed that NMES resistance training in the legs significantly lowered fasting blood glucose. Likewise, Griffin and colleagues [45] deployed 30 min of FES leg cycling during two to three weekly sessions for ten weeks on 18 individuals with chronic SCI. They found an improvement in glycemic response during oral glucose tolerance testing and reduced levels of inflammatory markers, c-reactive protein (CRP), interleukin-6 (IL-6), and tumor necrosis factor-α (TNF-α) [45].

**Table 1:** Effects of electrical stimulation exercise on cardiovascular and metabolic health.

Source	Participants	Treatments	Results
Farkas et al. [36]	*n* = 13 Chronic SCI	6—FES-LCE 5x/week for 16 weeks 7—ACE 5x/week for 16 weeks	FES +2.5% VO_2_peak ACE +20% VO_2_peak
Hasnan et al. [37]	*n* = 9 Chronic SCI	9—completed ACE, FES-LCE, FES-LCE, and outside hybrid cycling at 40%, 60%, and 80% of VO_2_peak.	FES-LCE + ACE and outside hybrid cycling resulted in significantly higher VO_2_peak, PO, and cardiac output than FES-LCE during all three submaximal intensities
Johnston et al. [39]	*n* = 30 Pediatric SCI (age 5–13)	10—FES-LCE 10—passive cycling 10—NMES	FES-LCE +16% VO_2_peak Passive cycling −27% VO_2_peak NMES −17% cholesterol level
Brurok et al. [40]	*n* = 6 Chronic SCI	6—hybrid HIIT-FES-LCE 3x/week for 8 weeks after a 7-week control period	+24% VO_2_peak +33% stroke volume Decreased cardiovascular disease risk
Hasnan et al. [41]	*n* = 8 Chronic SCI	8—hybrid HIIT FES-LCE + virtual reality 2–3 times per week (96 min per week)	+33% POpeak +20% VO_2_peak Blood lipids and glucose (no change)
Gorgey et al. [42]	*n* = 27 Chronic SCI	17—NMES-RT + FES-LCE 16-PMT + FES-LCE	NMES-RT + FES-LCE +29% VO_2_ PMT + FES-LCE +16% VO_2_
Griffin et al. [45]	*n* = 18 Chronic SCI	18—FES-LCE 2–3 times per week for 10 weeks	Cholesterol −1% Triglyceride −4% CRP −19% IL-6 −22% TNF-α −4% Insulin levels at 60 and 120 min during oral glucose tolerance test.
Gorgey et al. [43]	*n* = 9 Chronic SCI	11—NMES-RT 2 times/week for 16 weeks plus 2–6mg/day testosterone 11—testosterone only.	NMES grp Triglyceride −38% Cholesterol/HDL −14% Free fatty acids −24% Diet-only group Free fatty acids −20%

RT = resistance training; PO = power output; LCE = leg cycling exercise; FES-LCE = functional electrical stimulation leg cycling exercise; PMT = Passive Movement Therapy.

#### Summary

FES-LEC and ACE activities have been shown to provide cardiometabolic benefits; however, hybrid FES cycling activities, which combine both FES-LEC and ACE, have been found to be more beneficial for cardiometabolic health due to the engagement of more muscle activity and increased levels of exercise intensity. Eight weeks of thrice-weekly hybrid HIIT-FES cycling sessions showed increased stroke volume, cardiac output, and VO^2^peak levels. Combining NMES-RT and FES-LEC twice weekly has also been demonstrated to improve VO^2^peak levels, lower fasting blood glucose and improve cardiovascular blood markers. FES-LEC and NMES-RT have also been found to reduce inflammatory markers and improve glycemic control in middle-aged and elderly populations with type-2 diabetes, obesity, and SCI. More large-scale randomized control trials are needed to help confirm the findings of the current available evidence and to optimize the dose–response relative to the level of injury and the goals of individuals.

### 3.2. Muscle Strength and Mass (Table 2)

Roxley and colleagues [46] demonstrated the muscle-strengthening benefits of progressive resistance exercise combined with FES leg cycling. A 12-week randomized control trial on 28 individuals with incomplete SCIs combined 12 progressive resistance training sessions (knee extension and flexion, ankle dorsiflexion, and plantarflexion) and 24 FES leg cycling sessions, resulting in significantly greater quadricep and hamstring peak torque than a control group performing FES leg cycling without progressive resistance training. Moreover, the group that combined FES leg cycling with progressive resistance exercise demonstrated a more significant increase in muscle mass than the FES leg cycling-only group, 7% to 3%, respectively.

Gorgey et al. [42] also combined exercise protocols to optimize muscle hypertrophy. Twelve weeks of NMES resistance training twice weekly increased the cross-sectional area of the proximal, middle, and distal knee extensor muscle regions by 30–33%, 29–32%, and 26–28%, respectively. Furthermore, increases in knee extensor muscle hypertrophy were maintained by an additional twelve weeks of FES leg cycling.

Dolbow and associates [47] used HIIT-FES leg cycling to elicit positive body composition changes, including an increased leg lean mass of 7% and a decreased total body fat percentage of 2.5%. Five individuals with chronic SCIs performed HIIT-FES leg cycling thrice weekly for eight weeks with nutritional counseling one time per week and showed significantly greater improvements than the five-person control group that received nutritional counseling only.

While Farkas and colleagues [36] found only minimal non-significant increases in VO_2_peak after FES leg cycling five times per week for 16 weeks, there were greater body composition enhancements than ACE participants with a 4% increase in total body lean mass, a 7% increase in leg lean mass, and a decrease of 4% in total body fat percentage.

Speed of cadence has also been shown to affect gains in muscle mass. Seventeen individuals with SCIs were divided into the low-cadence and high-cycling-torque FES leg cycling group (20 revolutions per minute at 2.8 Nm) and the high cadence with low torque group (50 revolutions per minute at 0.8 Nm) for cycling sessions three times per week for six months. Both increased in muscle volume, with the low-cadence group having a significantly greater increase, 19% to 10%, respectively [48].

Gorgey and associates [27] combined NMES resistance training with dietary recommendations to demonstrate increases in thigh muscle mass. After 12 weeks of thrice-weekly NMES resistance training and diet, individuals with chronic SCIs observed increases in the whole-thigh cross-sectional area of 28%, the knee extensor cross-sectional area of 35%, and the knee flexor muscle cross-sectional area of 16%. In a separate study, Gorgey et al. [49] combined NMES resistance training twice weekly for 16 weeks with low-dose testosterone patches (2–6 mg per day). They again found significant increases in skeletal muscle cross-sectional area in the legs. Results from magnetic resonance images revealed a more than 20 cm^2^ increase in the whole-thigh muscle cross-sectional area and a 34% increase in the proximal region of the knee extensor muscle group, with a 32% increase for the middle knee extensor region and a 30% increase in the lower knee extension region. After accounting for intramuscular fat (IMF), the percentages increased to 43%, 34%, and 33%, respectively. Although the NMES resistance training concentrated on the knee extensors, the hip adductors and hamstring muscle groups also showed gains in cross-sectional areas. These gains were also accompanied by an increased basal metabolic rate, decreased visceral adipose tissue, and reduced inflammatory biomarkers [49].

NMES resistance training combined with testosterone has also been associated with a 29% fiber cross-sectional area and increased citrate synthase and succinate dehydrogenase. Surprisingly, the number of myonuclei increased following NMES resistance training and testosterone without successfully reporting fiber-type changes in histochemistry analysis via muscle biopsy [50,51].

The above findings suggested that the use of NMES resistance training with and without testosterone may promote health benefits and attenuate comorbidities in persons with SCIs. Furthermore, using NMES resistance training with relatively inexpensive, commercially available ankle weights may be as equally effective as using expensive FES leg cycling bikes for home use.

The evidence indicates that while both FES leg cycling and NMES resistance training can increase muscle mass, NMES resistance training outperforms FES leg cycling for producing muscle hypertrophy in individual muscle groups.

A recent systematic review indicated that there is conclusive evidence of the effects of electrical stimulation exercise on muscle size and lean mass. However, there is limited evidence to support the effects on percentage fat mass, regional fat mass, or ectopic adiposity following electrical stimulation exercise in persons with SCIs [52].

**Table 2:** Effects of electrical stimulation exercise on muscle strength and mass.

Source	Participants	Treatment	Results
Farkas et al. [36]	*n* = 13 chronic SCI	6—FES-LCE 5x/week for 16 weeks 7—ACE 5x/week for 16 weeks	FES +4% LM FES +7% legs LM ACE +2% LM FES −4% BF% ACE −5% BF%
Gorgey et al. [42]	*n* = 27 chronic SCI	17—NMES-RT + FES-LCE 16-PMT + FES-LCE	NMES-RT + FES-LCE +30–33% proximal Quadriceps CSA, 29–32% middle quadriceps CSA, 26–28% distal quadriceps CSA
Rosley et al. [46]	*n* = 23 chronic ‘incomplete’ SCI	10—FES-LCE + PRT 1 session PRT and 2 sessions FES-LCE weekly over 12 weeks 13—FES-LCE 3 sessions/weekly over 12 weeks	FES-LCE + PRT left hamstring peak torque +45% change, higher than FES-LCE FES-LCE + PRT right quadricep peak torque +31% change, greater than the FES-LCE FES-LCE + PRT Muscle volume +7% increase
Dolbow et al. [47]	*n* = 10 chronic SCI	5—interval HIIT-FES cycling 3x/week for 8 weeks and diet 5—diet alone	HIIT-FES cycling group Legs LM +7% Total BF% −2.5% Diet-only group No changes
Gorgey et al. [43]	*n* = 9 chronic SCI	11—NMES-RT 2 times/week for 16 weeks plus 2–6mg/day testosterone 11—testosterone only	NMES grp Thigh CSA +28% Knee ext CSA +35% Knee flexor +16%
Gorgey et al. [49]	*n*= 22 chronic SCI	11—NMES-RT 2 times/week for 16 weeks plus 2–6mg/day testosterone	NMES-RT Plus T grp (CSA) Prox knee ext +34% Mid knee ext +32% Low knee ext +30% -IMF Prox knee ext +43% Mid knee ext +34% Low knee ext +33%
Johnston et al. [39]	*n* = 17 chronic SCI	Low cadence 20 rpm High cadence 50 rpm 9—low cadence 8—high cadence 3x/week for 6 months	Low cadence +19% LM High cadence +10% LM

LM = lean mass; BF% = body fat percentage; CSA = cross-sectional area; PRT = progressive resistance training.

#### Summary

NMES-RT and FES-LEC have both been shown to be safe and effective ways to increase muscle mass and reduce body fat, with NMES-RT demonstrating a greater ability to increase the skeletal muscle cross-section area in the targeted muscles. Adding testosterone patches may also enhance the benefits. Twice-weekly sessions of NMES-RT for eight to twelve weeks has been found to be a successful regime, while thrice-weekly FES-LEC has also been successful. Adding progressive resistance exercise to FES-LEC has been shown to elevate benefits. HIIT-FES leg cycling, combined with nutritional counseling, has demonstrated potential for reducing body fat percentage. More research is required to determine optimal protocols regarding the type of electrical stimulation exercise to optimize the goals of those with SCIs and to determine at what stage the various protocols should be initiated in SCI recovery. 

### 3.3. Bone Mass (Table 3)

While evidence supports the concept that skeletal muscle hypertrophy can result from several weeks of FES exercise, slower bone metabolism typically requires at least six months to a year to produce improvements in bone health. Furthermore, positive bone health sequelae have not been consistent based on evidence. FES leg cycling and NMES resistance training provide only modest recovery or slowing of the rate of bone loss after an SCI [53].

Holman and associates [54] studied the effects of sixteen weeks of NMES resistance training on the legs along with receiving testosterone. Twenty men with SCIs were randomly placed in the NMES resistance training and testosterone group or the testosterone-only group. The effect sizes of changes in trabecular bone were estimated to be moderate in the proximal tibia and small in the distal femur. The authors speculated that these changes could increase significantly with more extended NMES resistance training and testosterone duration.

Frotzler et al. [55] had eleven individuals with SCIs perform FES leg cycling 3–4 times per week for a year, resulting in a 14% greater trabecular bone mineral density and a 7% increase in total bone mineral density in the distal femur. Similarly, Johnston and colleagues [19] demonstrated that using low-cadence FES leg cycling (20 revolutions per minute) three times per week for six months produced a 7% increase in trabecular bone. The largest positive impact on bone resulted from electrical stimulation at 1.5 times the body weight five times per week for two years, resulting in a 31% increase in bone mineral density in the distal tibia of individuals with SCIs [56].

Another study used the stimulation amplitude and the number of leg extension repetitions to highlight muscle and bone qualities in persons with SCIs. The authors noted that an arbitrary current of less than 100 mA and a leg extension repetition number greater than 70 out of 80 repetitions may suggest that persons with SCIs had greater muscle and bone qualities. The authors were capable of driving several regression equations to predict muscle size and knee bone mineral densities in persons with SCIs [57].

Available evidence suggests that the best results have been attained with FES or NMES leg exercises at least three times per week for several months to two years, with high-resistance exercises also necessary.

**Table 3:** Effects of electrical stimulation exercise on bone.

Source	Participants	Treatment	Results
Holman et al. [54]	*n* = 10 chronic SCIs	NMES-RT 2x/week	Distal femur—small trabecular increase Proximal tibia—medium trabecular increase
Frotzler et al. [55]	*n* = 11 chronic SCIs	FES-LCE 3–4x/week for 1 year	+14% BMD trabecular bone (distal femur) +7% BMD total bone (distal femur)
Shields and Dudley-Javorski, [56]	*n* = 7 (6 weeks post-SCI)	FES to plantar flexor muscles of one leg. The other leg was the control	+31% BMD (distal tibia)
Johnston et al. [48]	*n* = 17 chronic SCIs	Low cadence 20 rpm High cadence 50 rpm 9—low cadence 8—high cadence 3x/week for 6 months	+7% trabecular bone in distal femur

BMD = bone mineral density.

#### Summary

Changes in bone mass are much slower than muscle mass due to the relatively slow metabolic rate in skeletal bone. FES and NMES activities have been shown to provide a limited recovery of bone mass or decelerate the bone loss rate after an SCI. The current evidence shows that FES-LEC and NMES-RT programs require high-volume and high-intensity exercise to produce benefits in bone tissue. High-intensity exercise three to five times per week provides the best opportunity to slow bone loss or improve bone mineral density in individuals with SCIs. Training for at least six months to over a year may be required to achieve meaningful benefits. More research is needed to provide conclusive exercise guidelines for bone health after an SCI. Because of the limited benefits of electrical stimulation activities on bone health, future studies should focus on combining electrical stimulation exercises with bone maintenance medications or nutrition.

### 3.4. Diagnosis, Prognosis, and Treatment for Upper Limbs (Table 4)

**Figure 2:**
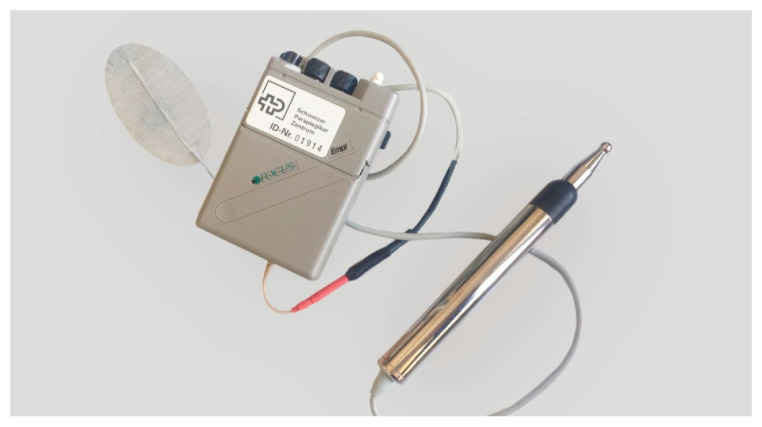
Nerve stimulator including pen electrode (active electrode) and self-adhesive electrode (reference electrode) for motor point mapping.

A further aspect of the application of electrical stimulation demonstrates the variety of its use, taking the upper extremities as an example in people with tetraplegia. Here, the application consists of a systematic diagnosis, prognosis, and treatment sequence. As previously published, the integrity of the lower motor neuron (LMN) can be tested by selectively assessing the upper limb muscles [58,59]. For this purpose, the muscles that are decisive for grasping and releasing objects are tested using a standardized measurement procedure employing electrical stimulation via a nerve, i.e., with a short pulse width. As the electrical excitability of nerve fibers (from 50 s = 0.05 ms) is earlier than that of muscle fibers (from 10 ms), the targeted stimulation of the motor points in the corresponding muscle can be used to determine whether an LMN lesion is present. This requires a reliable 2-channel stimulator that guarantees the output of the displayed intensity (amplitude mA) based on 250–300 μs (0.25–0.30 ms) with a frequency of 35 Hz. A pen electrode is recommended as the active electrode for higher precision (Figure 2).

The question of why this is ultimately important in treating the hands of people with tetraplegia is based on the fact that developing the tenodesis effect is still an essential aspect of upper-limb rehabilitation [60,61]. The tenodesis effect enables the affected person to grasp and release objects tentatively. Active dorsiflexion of the wrist leads to closure of the fist, which is achieved by passive insufficiency of the long finger flexors, which are positioned in approximation to provoke shortening. The hand is opened passively by relaxing the dorsiflexion, which consecutively leads to finger extension with volar flexion.

Clinical observations have shown that achieving this tenodesis effect is rarely successful in ensuring everyday functionality of the hand despite standardized positioning and appropriate splinting, including physio- and occupational therapy. Factors like edema, pre-existing contractures, and spasticity can influence the desired result. Another reason that should be considered is damage to the LMN on critical muscles that determine grasp and release. The key actuators are the extensor digitorum communis (EDC), the extensor pollicis longus (EPL), and the abductor pollicis longus (APL) for finger and thumb extension and the flexor digitorum profundus (FDP) and flexor pollicis longus (FPL) for flexion.

In a study involving 86 individuals with tetraplegia, it was shown that four different scenarios of hand forms develop, which have different innervation patterns regarding the LMN integrity of the critical muscles for hand opening and closing [62]. A subsequent investigation of the differently developing thumb positions, which also contribute significantly to the functionality of grasping and releasing, confirmed the findings previously obtained for the finger extensors and flexors [63].

In terms of hand form, the following four scenarios were identified:

1. The open flat hand, in which both the EDC and the FDP show LMN damage.

2. The hand that shows an incomplete tenodesis effect but with few functional limitations. In this case, the integrity of the LMN is preserved on both the EDC and FDP.

3. The classic hand with the well-functioning tenodesis effect, in which the EDC typically has a damaged LMN and the FDP an intact LMN.

4. The undesired claw hand, which is functionally unsuitable for manipulating objects. This is characterized by an intact LMN on the extensor side (EDC) and a damaged LMN on the flexor side (FDP).

This finding has implications for the treatment of the tetraplegic hand in rehabilitation. The use of electrical stimulation can be targeted based on the knowledge of the type of damage. In scenario 1, for example, where both the EDC and the FDP are denervated, long-pulse stimulation is indicated to prevent denervation atrophy, which results in the alteration of the muscle into connective and fatty tissue [64]. The likelihood of contractures developing is high.

**Figure 3:**
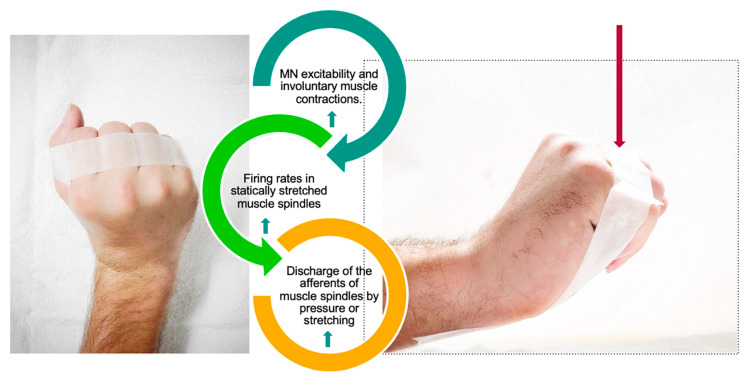
The impact on finger extensors by taping in the case of an intact LMN.

In the case of the claw hand described in scenario 4, the consequence in treatment is that classic taping of the hand to support the development of the tenodesis effect should preferably be avoided (Figure 3). Applying the stimulus via tape to the dorsal side of the fingers activates the muscle spindles. Muscle spindles are sensitive longitudinal traction receptors in skeletal muscle. Stretch-induced activation excites the Ia and II afferents in the spindle.

The discharge of the muscle spindle’s afferents depends on the muscle’s resting length. It can be increased by applying pressure to the muscle belly or tendon or by moving the joint in a direction that increases the stretch of the muscle.

In other words, taping the hand is counterproductive to developing a tenodesis effect [65].

The effective and efficient electrical stimulation of the various neurologically damaged muscles of the upper limb is essential for successful treatment. Electrical stimulation can be used as a diagnostic tool to determine the damage. Applied and used promptly following an SCI, it allows a prediction about the development of hand function [32].

**Table 4:** Use of electrical stimulation for diagnosis, prognosis, and treatment for upper limbs.

Source	Participants	Testing or Treatment	Results
Bersch et al. [58]	*n* = 32 Tetraplegia	Retrospective analysis Defined motor points and wrist and finger activities to detect UMN/LMN Lesions	16 hands developed tenodesis grasp all with LMN of EDC 24: no tightness of finger flexors
Bersch et al. [59]	*n* = 24 Tetraplegia	Tested forearms for LMN/UMN lesions 44 arms analyzed	FDPIII—26 arms with UMN lesion 10 arms with partial denervation 5 arms with denervation FPL—16 arms with UMN lesion 12 arms with partial denervation 14 arms with denervation
Jung et al. [60]	*n* = 37 Tetraplegia (preserved wrist extension with paralysis in fingers)	Assessment of passive tenodesis grasp (open and closed) GRASSP testing	Those with 4–5/5 muscle strength showed higher GRASSP scores than those with 3/5 wrist extension
Bersch et al. [62]	*n* = 220 Tetraplegia Data Base	Retrospective analysis of AIS and MMT of arm and hand muscles at different time points	Hand and arm function predicted by MP and AIS and used as the basis for providing an individualized treatment plan
Koch-Boner et al. [63]	*n* = 82 159 hands Tetraplegia	Divided into 3 thumb positions (key pinch, slack thumb, and thumb-in-palm). Muscle testing and motor point testing	Muscles showed a different expression of MP and the MMT values between key punch and thumb slack positions. MMT of FPL was greater in the group “thumb-in-palm” compared with the “key pinch” position
Bersch & Friden [64]	*n* = 22 Tetraplegia ECU and 1st dorsal interosseous denervated	Electrical stimulation 33 min, 5x/wk, 12 wks	ECU +27% muscle thickness +71% pennation angle 1st dorsal interosseus +46% muscle thickness +100% pennation angle

GRASSP = Graded and Redefined Assessment of Strength, Sensibility, and Prehension.

#### Summary

The electrical stimulation testing of upper-extremity muscles can provide diagnostic information regarding upper or lower motor neuron injury to muscles that are key to upper-extremity function. This information can also be used to determine the prognosis of possible future deformities of the hands and how to best approach rehabilitation to achieve the tenodesis effect for grasping and overall functional recovery as well as reconstructive surgery, including muscle-tendon and nerve transfers. The research is extensive and detailed in this area, with guidelines that can help provide targeted electrical stimulation exercises to help decrease the risk of contractures and improve the recovery of hand function. Further research is required to determine the optimal dose–response effects of electrical stimulation training on injuries of varying levels and degrees of completeness.

## 4. Conclusions

Overall, electrical stimulation activities are safe and effective methods for exercise (NMES and FES) and testing for motor neuron lesions in individuals with SCIs and other paralytic or paretic conditions. They should be considered part of a comprehensive rehabilitation program in diagnosing, prognosing, and treating individuals with SCIs to improve function, physical activity, and overall health.

## Data Availability

Not applicable.

## References

[1] Deley G., Denuziller J., Babault N. (2015). Functional electrical stimulation: Cardiorespiratory adaptations and applications for training in paraplegia. Sports Med..

[2] Phillips A.A., Krassioukov A.V. (2015). Contemporary cardiovascular concerns after spinal cord injury: Mechanisms, maladaptations, and management. J. Neurotrauma.

[3] Ginis K.A.M., Latimer A.E., Arbour-Nicitopoulos K.P., Buchholz A.C., Bray S.R., Craven B.C., Hayes K.C., Hicks A.L., McColl M.A., Potter P.J. (2010). Leisure time physical activity in a population-based sample of people with spinal cord injury part I: Demographic and injury-related correlates. Arch. Phys. Med. Rehabil..

[4] Colley R.C., Garriguet D., Janssen I., Craig C.L., Clarke J., Tremblay M.S. (2011). Physical activity of Canadian adults: Accelerometer results from the 2007 to 2009 Canadian Health Measures Survey. Health Rep..

[5] Soriano J.E., Squair J.W., Cragg J.J., Thompson J., Sanguinetti R., Vaseghi B., Emery C.A., Grant C., Charbonneau R., Larkin-Kaiser K.A. (2022). A national survey of physical activity after spinal cord injury. Sci. Rep..

[6] Guest J., Datta N., Jimsheleishvili G., Gater D.R. (2022). Pathophysiology, Classification and Comorbidities after Traumatic Spinal Cord Injury. J. Pers. Med..

[7] Gater D.R., Farkas G.J., Tiozzo E. (2021). Pathophysiology of neurogenic obesity after spinal cord injury. Top. Spinal Cord Inj. Rehabil..

[8] Farkas G.J., Gater D.R. (2018). Neurogenic obesity and systemic inflammation following spinal cord injury: A review. J. Spinal Cord Med..

[9] Gordon P.S., Farkas G.J., Gater D.R. (2021). Neurogenic Obesity-Induced Insulin Resistance and Type 2 Diabetes Mellitus in Chronic Spinal Cord Injury. Top. Spinal Cord Inj. Rehabil..

[10] Nash M.S., Groah S.L., Gater D.R., Dyson-Hudson T.A., Lieberman J.A., Myers J., Sabharwal S., Taylor A.J. (2019). Identification and management of cardiometabolic risk after spinal cord injury. J. Spinal Cord Med..

[11] Shin J.W., Kim T., Lee B.S., Kim O. (2022). Factors Affecting Metabolic Syndrome in Individuals with Chronic Spinal Cord Injury. Ann. Rehabil. Med..

[12] Faghri P.D., Yount J.P., Pesce W.J., Seetharama S., Votto J.J. (2001). Circulatory hypokinesis and functional electric stimulation during standing in persons with spinal cord injury. Arch. Phys. Med. Rehabil..

[13] Furlan J.C., Fehlings M.G. (2008). Cardiovascular complications after acute spinal cord injury: Pathophysiology, diagnosis, and management. Neurosurg. Focus.

[14] Wecht J.M., Harel N.Y., Guest J., Kirshblum S.C., Forrest G.F., Bloom O., Ovechkin A.V., Harkema S. (2020). Cardiovascular autonomic dysfunction in spinal cord injury: Epidemiology, diagnosis, and management. Semin. Neurol..

[15] Eschlböck S., Wenning G., Fanciulli A. (2017). Evidence-based treatment of neurogenic orthostatic hypotension and related symptoms. J. Neural Transm..

[16] Mathias C.J. (2006). Orthostatic hypotension and paroxysmal hypertension in humans with high spinal cord injury. Prog. Brain Res..

[17] Krassioukov A., Linsenmeyer T.A., Beck L.A., Elliott S., Gorman P., Kirshblum S., Vogel L., Wecht J., Clay S. (2021). Evaluation and management of autonomic dysreflexia and other autonomic dysfunctions: Preventing the highs and lows: Management of blood pressure, sweating, and temperature dysfunction. Top. Spinal Cord Inj. Rehabil..

[18] Eldahan K.C., Rabchevsky A.G. (2018). Autonomic dysreflexia after spinal cord injury: Systemic pathophysiology and methods of management. Auton. Neurosci..

[19] Tweedy S.M., Beckman E.M., Geraghty T.J., Theisen D., Perret C., A Harvey L., Vanlandewijck Y.C. (2017). Exercise and sports science Australia (ESSA) position statement on exercise and spinal cord injury. J. Sci. Med. Sport.

[20] Nash M.S., Bilzon J.L.J. (2018). Guideline Approaches for Cardioendocrine Disease Surveillance and Treatment Following Spinal Cord Injury. Curr. Phys. Med. Rehabil. Rep..

[21] Cowan R.E., Nash M.S., Anderson-Erisman K. (2012). Perceived exercise barriers and odds of exercise participation among persons with SCI living in high-income households. Top. Spinal Cord Inj. Rehabil..

[22] Luo S., Xu H., Zuo Y., Liu X., All A.H. (2020). A Review of Functional Electrical Stimulation Treatment in Spinal Cord Injury. Neuromolecular Med..

[23] Sadowsky C.L., Hammond E.R., Strohl A.B., Commean P.K., Eby S.A., Damiano D.L., Wingert J.R., Bae K.T., McDonald J.W. (2013). Lower extremity functional electrical stimulation cycling promotes physical and functional recovery in chronic spinal cord injury. J. Spinal Cord Med..

[24] Karamian B.A., Siegel N., Nourie B., Serruya M.D., Heary R.F., Harrop J.S., Vaccaro A.R. (2022). The role of electrical stimulation for rehabilitation and regeneration after spinal cord injury. J. Orthop. Traumatol..

[25] Hamid S., Hayek R. (2008). Role of electrical stimulation for rehabilitation and regeneration after spinal cord injury: An overview. Eur. Spine J..

[26] Bochkezanian V., Newton R.U., Trajano G.S., Blazevich A.J. (2018). Effects of Neuromuscular Electrical Stimulation in People with Spinal Cord Injury. Med. Sci. Sports Exerc..

[27] Dudley-Javoroski S., Shields R.K. (2008). Muscle and bone plasticity after spinal cord injury: Review of adaptations to disuse and to electrical muscle stimulation. J. Rehabil. Res. Dev..

[28] Mahoney E.T., Bickel C.S., Elder C., Black C., Slade J.M., Apple D., Dudley G.A. (2005). Changes in skeletal muscle size and glucose tolerance with electrically stimulated resistance training in subjects with chronic spinal cord injury. Arch. Phys. Med. Rehabil..

[29] de Freitas G.R., Szpoganicz C., Ilha J. (2018). Does Neuromuscular Electrical Stimulation Therapy Increase Voluntary Muscle Strength After Spinal Cord Injury? A Systematic Review. Top. Spinal Cord Inj. Rehabil..

[30] Atkins K.D., Bickel C.S. (2021). Effects of functional electrical stimulation on muscle health after spinal cord injury. Curr. Opin. Pharmacol..

[31] Bickel C.S., Yarar-Fisher C., Mahoney E.T., McCully K.K. (2015). Neuromuscular Electrical Stimulation-Induced Resistance Training After SCI: A Review of the Dudley Protocol. Top. Spinal Cord Inj. Rehabil..

[32] Ho C.H., Triolo R.J., Elias A.L., Kilgore K.L., DiMarco A.F., Bogie K., Vette A.H., Audu M.L., Kobetic R., Chang S.R. (2014). Functional electrical stimulation and spinal cord injury. Phys. Med. Rehabil. Clin. N. Am..

[33] Lu X., Battistuzzo C.R., Zoghi M., Galea M.P. (2015). Effects of training on upper limb function after cervical spinal cord injury: A systematic review. Clin. Rehabil..

[34] van der Scheer J.W., Goosey-Tolfrey V.L., Valentino S.E., Davis G.M., Ho C.H. (2021). Functional electrical stimulation cycling exercise after spinal cord injury: A systematic review of health and fitness-related outcomes. J. NeuroEng. Rehabil..

[35] Street T., Davis G.M. (2022). New Clinical Practice Guidelines for Functional Electrical Stimulation (FES) Cycling and Walking. Forward.

[36] Farkas G.J., Gorgey A.S., Dolbow D.R., Berg A.S., Gater D.R. (2021). Energy expenditure, cardiorespiratory fitness, and body composition following arm cycling or functional electrical stimulation exercises in spinal cord injury: A 16-week randomized controlled trial. Top. Spinal Cord Inj. Rehabil..

[37] Hasnan N., Ektas N., Tanhoffer A.I., Tanhoffer R., Fornusek C., Middleton J.W., Husain R., Davis G.M. (2013). Exercise responses during functional electrical stimulation cycling in individuals with spinal cord injury. Med. Sci. Sports Exerc..

[38] Davis G.M. (2023). RehabWeek, Presentation. Discipline of Exercise and Sport Sciences, Sydney School of Health Sciences, Faculty of Medicine and Health, The University of Sydney, Camperdown, NSW 2006, Australia.

[39] Johnston T.E., Smith B.T., Mulcahey M.J., Betz R.R., Lauer R.T. (2009). A randomized controlled trial on the effects of cycling with and without electrical stimulation on cardiorespiratory and vascular health in children with spinal cord injury. Arch. Phys. Med. Rehabil..

[40] Brurok B., Helgerud J., Karlsen T., Leivseth G., Hoff J. (2011). Effect of aerobic high-intensity hybrid training on stroke volume and peak oxygen consumption in men with spinal cord injury. Am. J. Phys. Med. Rehabil..

[41] Hasnan N., Engkasan J.P., Husain R., Davis G.M. (2013). High-Intensity Virtual-reality Arm plus FES-leg Interval Training in Individuals with Spinal Cord Injury. Biomed. Tech..

[42] Gorgey A.S., Khalil R.E., Carter W., Ballance B., Gill R., Khan R., Goetz L., Lavis T., Sima A.P., Adler R.A. (2023). Effects of two different paradigms of electrical stimulation exercise on cardio-metabolic risk factors after spinal cord injury. A randomized clinical trial. Front. Neurol..

[43] Gorgey A.S., Mather K.J., Cupp H.R., Gater D.R. (2012). Effects of resistance training on adiposity and metabolism after spinal cord injury. Med. Sci. Sports Exerc..

[44] Sanchez M.J., Mossayebi A., Sigaroodi S., Apaflo J.N., Galvan M.J., Min K., Agullo F.J., Wagler A., Bajpeyi S. (2023). Effects of neuromuscular electrical stimulation on glycemic control: A systematic review and meta-analysis. Front. Endocrinol..

[45] Griffin L., Decker M.J., Hwang J.Y., Wang B., Kitchen K., Ding Z., Ivy J. (2009). Functional electrical stimulation cycling improves body composition, metabolic and neural factors in persons with spinal cord injury. J. Electromyogr. Kinesiol..

[46] Rosley N., Hasnan N., Hamzaid N.A., Davis G.M., Manaf H. (2022). Effects of a combined progressive resistance training and functional electrical stimulation-evoked cycling exercise on lower limb muscle strength of individuals with incomplete spinal cord injury: A randomized controlled study. Turk. J. Phys. Med. Rehabil..

[47] Dolbow D.R., Credeur D.P., Lemacks J.L., Stokic D.S., Pattanaik S., Corbin G.N., Courtner A.S. (2021). Electrically induced cycling and nutritional counseling for counteracting obesity after spinal cord injury: A pilot study. J. Spinal Cord Med..

[48] Johnston T.E., Marino R.J., Oleson C.V., Schmidt-Read M., Benjamin E., Leiby B.E., Sendecki J., Modlesky C.M. (2016). Musculoskeletal effects of 2 functional electrical stimulation cycling paradigms conducted at different cadences for people with spinal cord injury: A pilot study. Arch. Phys. Med. Rehabil..

[49] Gorgey A.S., Khalil R.E., Gill R., Gater D.R., Lavis T.D., Cardozo C.P., Adler R.A. (2019). Low-dose testosterone and evoked resistance exercise after spinal cord injury on cardio-metabolic risk factors: An open-label randomized clinical trial. J. Neurotrauma.

[50] Gorgey A.S., Graham Z.A., Chen Q., Rivers J.F., Adler R.A., Lesnefsky E.J., Cardozo C.P. (2020). Sixteen weeks oftestosterone with or without evoked resistance training on protein expression, fiber hypertrophy and mitochondrial health after spinal cord injury. J. Appl. Physiol..

[51] Lai R.E., Gorgey A.S. (2021). Low-dose testosterone replacement therapy and electrically evoked resistance training enhance muscle quality after spinal cord injury. Neural Regen. Res..

[52] Bekhet A.H., Jahan A.M., Bochkezanian V., Musselman K.E., Elsareih A.A., Gorgey A.S. (2022). Effects of Electrical Stimulation Training on Body Composition Parameters After Spinal Cord Injury: A Systematic Review. Arch. Phys. Med. Rehabil..

[53] Sutor T.W., Kura J., Mattingly A.J., Otzel D.M., Yarrow J.F. (2022). The Effects of Exercise and Activity-Based Physical Therapy on Bone after Spinal Cord Injury. Int. J. Mol. Sci..

[54] Holman M.E., Chang G., Ghatas M.P., Saha P.K., Zhang X., Khan M.R., Sima A.P., Adler R.A., Gorgey A.S. (2021). Bone and non-contractile soft tissue changes following open kinetic chain resistance training and testosterone treatment in spinal cord injury: An exploratory study. Osteoporos. Int..

[55] Frotzler A., Coupaud S., Perret C., Kakebeeke T.H., Hunt K.J., Donaldson N.d.N., Eser P. (2008). High-volume FES-cycling partially reverses bone loss in people with chronic spinal cord injury. Bone.

[56] Shields R.K., Dudley-Javoroski S., Petrie M., Suneja M., Littmann A.E., Iguchi M., DiMarco A.F., Kowalski K.E., Harris R.L.W., Putman C.T. (2006). Musculoskeletal plasticity after acute spinal cord injury: Effects of long-term neuromuscular electrical stimulation training. J. Neurophysiol..

[57] Gorgey A.S., Khalil R.E., Sutor T.W., Goldsmith J.A., Cifu D.X. (2022). Employment of Neuromuscular Electrical Stimulation to Examine Muscle and Bone Qualities after Spinal Cord Injury. J. Clin. Med..

[58] Bersch I., Koch-Borner S., Fridén J. (2018). Electrical stimulation-a mapping system for hand dysfunction in tetraplegia. Spinal Cord.

[59] Bersch I., Koch-Borner S., Fridén J. (2020). Motor Point Topography of Fundamental Grip Actuators in Tetraplegia: Implications in Nerve Transfer Surgery. J. Neurotrauma.

[60] Jung H.Y., Lee J., Shin H.I. (2018). The natural course of passive tenodesis grip in individuals with spinal cord injury with preserved wrist extension power but paralyzed fingers and thumbs. Spinal Cord.

[61] Johanson M.E., Murray W.M. (2002). The unoperated hand: The role of passive forces in hand function after tetraplegia. Hand Clin..

[62] Bersch I., Krebs J., Fridén J. (2022). A Prediction Model for Various Treatment Pathways of Upper Extremity in Tetraplegia. Front. Rehabil. Sci..

[63] Koch-Borner S., Bersch U., Grether S., Fridén J., Schibli S., Bersch I. (2023). Different thumb positions in the tetraplegic hand. Arch. Phys. Med. Rehabil..

[64] Bersch I., Fridén J. (2021). Electrical stimulation alters muscle morphological properties in denervated upper limb muscles. Ebiomedicine.

[65] Zijdewind I., Thomas C.K. (2003). Motor unit firing during and after voluntary contractions of human thenar muscles weakened by spinal cord injury. J. Neurophysiol..

